# Protein Glycosylation and Tumor Microenvironment Alterations Driving Cancer Hallmarks

**DOI:** 10.3389/fonc.2019.00380

**Published:** 2019-05-14

**Authors:** Andreia Peixoto, Marta Relvas-Santos, Rita Azevedo, Lúcio Lara Santos, José Alexandre Ferreira

**Affiliations:** ^1^Experimental Pathology and Therapeutics Group, Portuguese Institute of Oncology, Porto, Portugal; ^2^Institute of Biomedical Sciences Abel Salazar, University of Porto, Porto, Portugal; ^3^Tumour and Microenvironment Interactions Group, INEB-Institute for Biomedical Engineering, Porto, Portugal; ^4^Instituto de Investigação e Inovação em Saúde, Universidade do Porto, Porto, Portugal; ^5^Department of Surgical Oncology, Portuguese Institute of Oncology, Porto, Portugal; ^6^Porto Comprehensive Cancer Center, Porto, Portugal

**Keywords:** cancer, microenvironment, glycans, protein glycosylation, cancer hallmarks

## Abstract

Decades of research have disclosed a plethora of alterations in protein glycosylation that decisively impact in all stages of disease and ultimately contribute to more aggressive cell phenotypes. The biosynthesis of cancer-associated glycans and its reflection in the glycoproteome is driven by microenvironmental cues and these events act synergistically toward disease evolution. Such intricate crosstalk provides the molecular foundations for the activation of relevant oncogenic pathways and leads to functional alterations driving invasion and disease dissemination. However, it also provides an important source of relevant glyco(neo)epitopes holding tremendous potential for clinical intervention. Therefore, we highlight the transversal nature of glycans throughout the currently accepted cancer hallmarks, with emphasis on the crosstalk between glycans and the tumor microenvironment stromal components. Focus is also set on the pressing need to include glycans and glycoconjugates in comprehensive panomics models envisaging molecular-based precision medicine capable of improving patient care. We foresee that this may provide the necessary rationale for more comprehensive studies and molecular-based intervention.

## Introduction

Genetic and epigenetic alterations are considered primary causes of cancer development, with downstream phenotypic changes at the protein level being amongst the driving forces of cancer progression and dissemination. Specifically, post-translational modifications, as glycosylation, impact on protein trafficking, stability and folding, ultimately altering its biochemical, and biophysical properties (1, 2). Moreover, glycans dictate proteolysis patterns and directly mediate ligand-receptor interactions, oncogenic signaling transduction, immune recognition, migration and both cell-cell and cell-matrix adhesion (3–5). In addition, intracellular *O*-GlcNAc glycosylation (in Ser/Thr residues) of proteins plays a major role in cell physiology and signaling by direct competition with phosphorylation (6). As such, several studies have so far disclosed a plethora of glycans that confer selective advantage to tumor cells, while providing important surrogate biomarkers for specific biological milieus (7, 8). Moreover, while there are few evidences of mutations in genes involved in glycosylation pathways, it is well known that transcriptional and metabolic reprograming of cancer cells has tremendous impact on their glycome and glycoproteome, leading not only to the overexpression but also to the *de novo* expression of specific glycoepitopes (9, 10). Despite its sour side, cancer-specific alterations in protein glycosylation provide a unique opportunity for clinical intervention. The uniqueness of the created molecular features may be explored to selectively target tumor cells or may provide non-invasive biomarkers after secretion or shedding into body fluids from tumor sites (11, 12).

Building on these findings, the glycobiology field has been progressing toward a more functional understanding of glycosylation impact on cancer biology, disease progression, and dissemination. While specific details on the biosynthesis and diversity of cancer-associated glycans may be found in recent reviews (7, 8), the following sections attempts to highlight the transversal nature of glycans, glycoproteins, and glycan-binding proteins throughout currently accepted cancer hallmarks, with emphasis on the crosstalk between glycans and the stromal components of the tumor microenvironment (**Figure 2**). These comprehend: (i) sustained proliferative signaling; (ii) resistance to cell death; (iii) deregulated cellular energetics; (iv) evasion of growth suppressors; (v) genome instability and mutations; (vi) replicative immortality; (vii) induction of angiogenesis; (viii) activation of invasion and metastasis; (ix) tumor-promoting inflammation; and (x) immune escape (13). Moreover, we highlight the significance of the most promising protein glycosignatures in cancer arising from the cancer cells-microenvironment crosstalk, its relevance and main milestones facing clinical translation and personalized medicine, as well as the opportunities provided by high-throughput glycomics and glycoproteomics toward molecular-based precision oncology. We foresee that this may provide the necessary rationale for more comprehensive studies and molecular-based intervention.

## Protein Glycosylation in Cancer

Glycosylation is the most common, structurally diverse and complex posttranslational modification of membrane-bound proteins, being a non-templated but highly regulated process that rapidly changes in response to physiological and pathological contexts. Glycans result from the highly coordinated action of nucleotide sugar transporters, glycosyltransferases (GTs) and glycosidases in the endoplasmic reticulum (ER) and Golgi apparatus (GA). Two main classes of glycans can be found in membrane and extracellular glycoproteins: (i) *O*-GalNAc glycans, initiated in the GA by the attachment of a GalNAc to the hydroxyl groups of serine (Ser) or threonine (Thr) residues, forming the simplest *O*-glycan Tn antigen (GalNAcα-Ser/Thr). The Tn antigen may be further elongated into different core structures that serve as scaffolds for more complex *O*-GalNAc glycans; (ii) *N*-glycans, whose biosynthesis starts in the ER with the addition of an oligosaccharide chain to an asparagine (Asn) residue in a peptide consensus sequence of Asn-X-Ser/Thr (X denotes any amino acid except proline). *N*-glycans experience further structural maturation in the GA to yield either partially unprocessed oligomannose antenna or, more frequently, complex or hybrid type structures, which frequently experience further elongation. Both *O*- and *N*-glycan chains are generally branched and/or elongated and may present sialic acids, Lewis blood group related antigens or ABO(H) blood group determinants as terminal structures (8). Further glycan diversity results from several modifications in individual sugars, including *O*-Acetylation of sialic acids and *O*-Sulfation of galactose and *N*-acetylglucosamine residues. Mature glycans may still experience structural remodeling at the cell-surface by extracellular glycosyltransferases and glycosidases freely circulating in the plasma or carried by platelets, further increasing the glycome's structural complexity and dynamic nature (14–16). In addition, other less abundant and far less studied classes of protein glycans can be found at the cell membrane, including *O*-Fucosylation, *O*-Mannosylation, *O*-glucosylation, and *C*-Mannosylation (17–19). This provides a wide array of potential posttranslational modifications that decisively contribute to define protein functional roles.

In addition to the structural modification of extracellular and cell membrane proteins, intracellular proteins can also be glycosylated with functional implications. Namely, intracellular glycosylation results from the reversible attachment of a *N*-acetylglucosamine moiety (β-linked GlcNAc) to Ser or Thr residues in cytoplasmic and nuclear proteins (20–22). The GlcNAc residue is generally not elongated or modified to generate complex structures (23). The dynamic cycling of *O*-GlcNAcylation is catalyzed by two ubiquitously expressed and highly conserved enzymes: uridine diphospho-*N*-acetylglucosamine:polypeptide β-*N*-acetylglucosaminyltransferase (*O*-GlcNAc transferase, OGT), which adds GlcNAc to the hydroxyl side chain of Ser and Thr, and *N*-acetyl-β-D-glucosaminidase (*O*-GlcNAcase, OGA), the enzyme that removes *O*-GlcNAc. This posttranslational modification has regulatory functions akin to phosphorylation, modulating protein conformation, stability, and reversible multimeric protein assembly (24). Moreover, it functions as a nutrient sensor, providing a biochemical switch to enable the cell adaptation to glucose level alterations and hormonal cues, while regulating a myriad of cellular processes like cellular adhesion, DNA transcription, translation, nuclear transport, and cytoskeletal assembly (25, 26). Interestingly; different isoforms of OGT and OGA vary in length and subcellular localization, suggesting that they target distinct subsets of the proteome (27).

**Figure 1:**
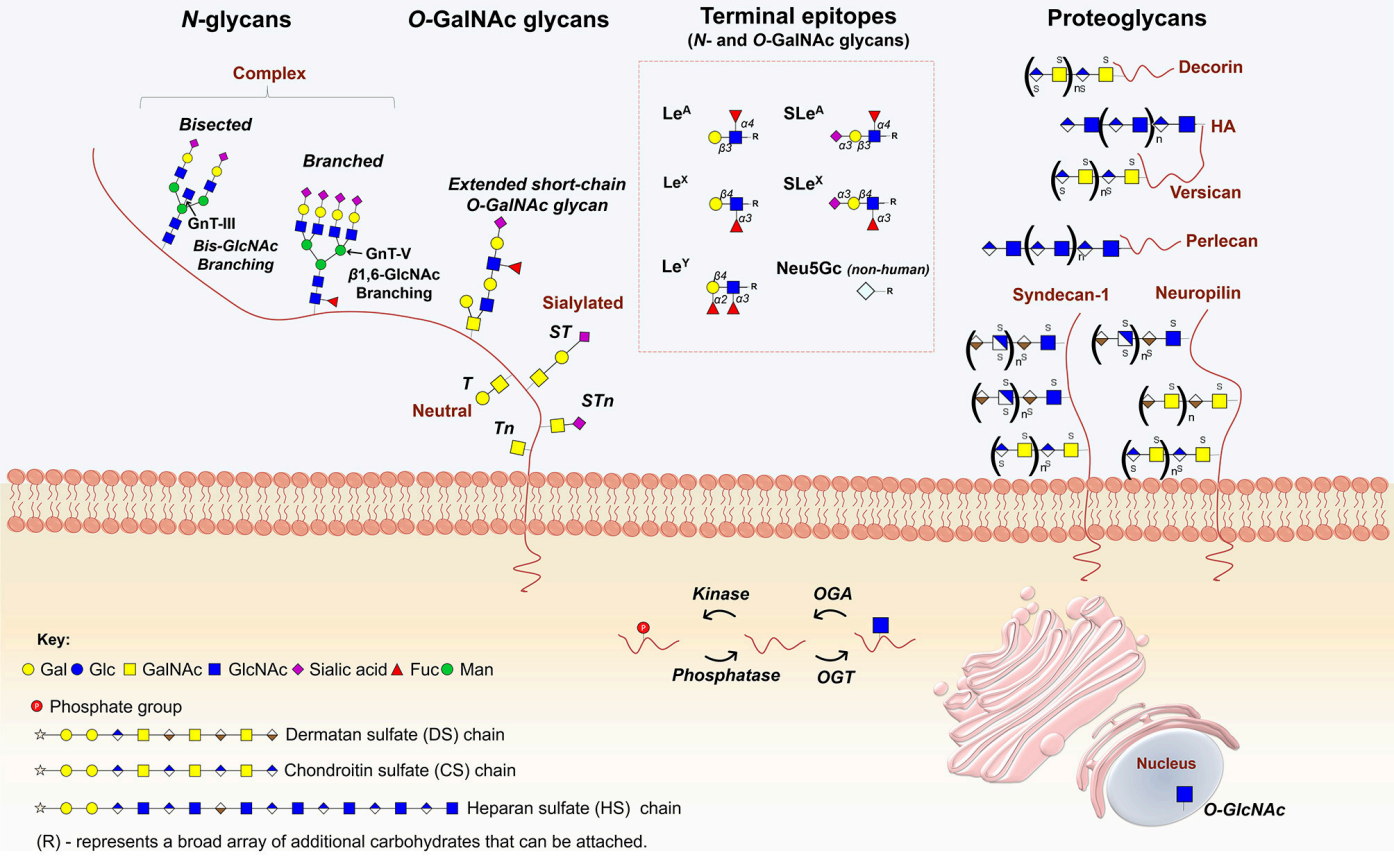
Main classes of glycans modulating cancer hallmarks. *N*-glycans, whose biosynthesis starts in the endoplasmic reticulum (ER) with the addition of an oligosaccharide chain to an asparagine (Asn) residue, experience further structural maturation in the golgi apparatus (GA) to yield complex bisected and branched structures. *O*-GalNAc glycans, initiated in the GA by the attachment of a GalNAc to the hydroxyl groups of serine (Ser) or threonine (Thr) residues, forming the simplest *O*-glycan Tn antigen (GalNAcα-Ser/Thr), may be further elongated into different core structures that serve as scaffolds for more complex *O*-GalNAc glycans. Both *O*- and *N*-glycan chains are generally branched and/or elongated and may present sialic acids, Lewis blood group related antigens and/or their sialylated counterparts as terminal structures. Proteoglycans constitute another class of functionally complex glycoconjugates found as transmembrane, basement membrane and extracellular matrix (ECM) components, exhibiting one or several high molecular weight glycosaminoglycan (GAG) chains covalently attached to a protein core. The figure highlights the structures of some of the most relevant glycans and glycoconjugates driving cancer hallmarks.

It has been long known that advanced tumors present severe dysregulations in glycosylation pathways, with tumor-associated carbohydrates arising from incomplete or neo-synthesis processes (28). Of note, incomplete synthesis originating truncated structures is more common in early carcinogenesis (29, 30), while the *de novo* synthesis of neoantigens is more frequent in advanced stages of several cancers (31). The most reported alterations associated to cancer include the over- and/or *de novo* expression of short-chain *O*-GalNAc glycans (Tn, T, Sialyl-T, and Sialyl-Tn), Lewis blood group related antigens and their sialylated counterparts [sialyl-Lewis A (SLe^a^) and X (SLe^x^)], as well as complex branched *N*-glycans (32–34) (Figure 1). Many of these structural features are common to most advanced solid tumors and often associate with poor prognosis, suggesting common molecular mechanisms, which is yet to be proven. Nevertheless, distinct proteome signatures, glycosylation density, and glycosite distribution may ultimately dictate organ, cell-type and cancer-specific molecular signatures and clinically relevant glycoforms.

Another class of cell-surface glycoconjugates that populate the cell surface and extracellular matrix are proteoglycans, generally composed of one or several high molecular weight glycosaminoglycan (GAG) chains, and composed of sulphated disaccharide repeating units of chondroitin sulfate (CS), heparan sulfate (HS), or dermatan sulfate (DS) covalently attached to a protein core (Figure 1). These polymers can be found as transmembrane, basement membrane and extracellular matrix (ECM) components, presenting high affinities for various ECM constituents and cell adhesion molecules. As such, proteoglycans largely contribute to the acquisition of cancer hallmarks by playing a role in intercellular and ECM interactions, as well as in cellular signaling, especially as co-receptors for growth factors and tyrosine kinase receptors (35, 36).

Overall, the most widely occurring glycosylation modifications in cancer stem from alterations in glycan length, often toward shorter *O*-glycans and more branched *N*-glycans. This is accompanied by critical changes in glycans sialylation and fucosylation that impact on the nature of terminal epitopes at glycan chains. In addition, several changes in glycan chains have been reported for glycosaminoglycans (GAG). The structural nature of glycan alteration in cancer and underlying biosynthesis mechanisms have been comprehensively reviewed in recent years (7, 8, 37) and will not be covered in detail here. Aberrant glycosylation actively contributes to tumor progression by regulating tumor proliferation, invasion, metastasis, and angiogenesis (7, 38), being frequently cited as a hallmark of cancer (39). As such, we reinforce this notion by highlighting aberrant glycosylation as an integral part of all recognized cancer hallmark traits. Furthermore, we include the cabal contribution of stromal cells and microenvironmental features for tumor progression and aggressiveness.

## Tumor Microenvironment and Glycosylation Crosstalk Toward the Hallmarks of Cancer

The glycocalyx, combining glycoproteins and sugar moieties located on the external side of the plasma membrane, drives the interplay between cancer cells and the tumor microenvironment (TME), a complex scaffold of extracellular matrix (ECM) and various cell types. Both glycans, glycoconjugates and the TME actively contribute to the acquisition of cancer hallmarks, adding another dimension of complexity to cancer progression by influencing cell adhesion and cell-cell recognition, as well as intracellular signaling and ECM interactions (8, 40, 41). Herein, we will highlight the glycosylation-mediated promotion of cancer hallmarks, including the role of stromal cells.

### Sustained Proliferative Signaling

**Figure 2:**
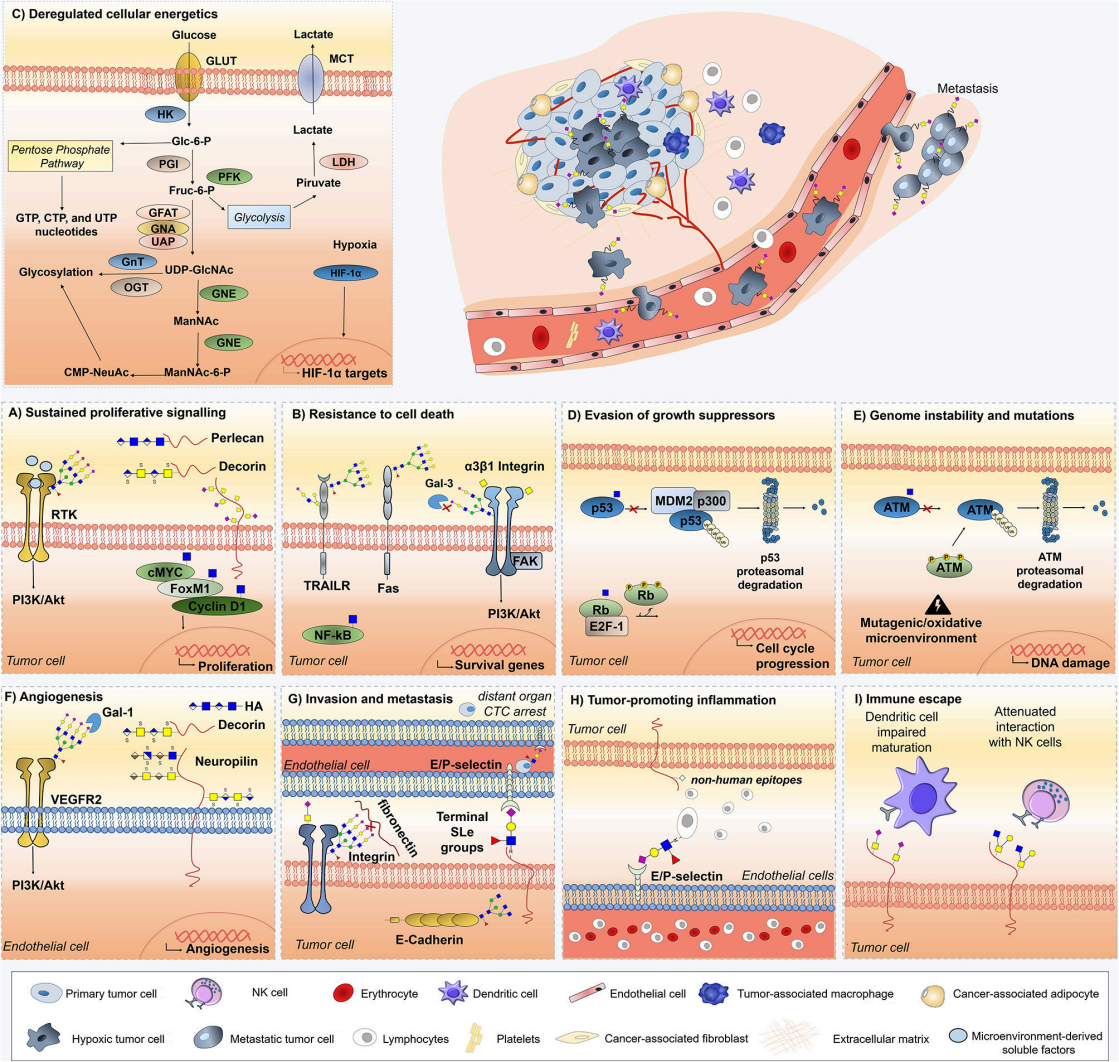
Role of glycans, glycoproteins, glycan-binding proteins, and proteoglycans across currently accepted cancer hallmarks. Glycans (sTn, sLe^A/X^, Neu5Gc,β1,6-branched *N*-glycans), glycoproteins (Fas, TRAIL-R, integrin α3β1, VEGFR2, ATM, p53, Rb), proteoglycans (decorin, neuropilin-1,-2, hyaluronic acid, versican, perlecan, hyaluronic acid), lectins (Gal-3 and Gal-1), and *O*-GlcNAcylated transcription factors (c-Myc, Fox M1, cyclin D1, NF-κB) are mechanistically implicated in cancer hallmarks acquisition and thus represented. Overall, the illustrations focus on particular molecular mechanisms driving hallmark acquisition; namely **(A)** sustaining proliferative signaling, **(B)** resistance to cell death, **(C)** deregulated cellular energetics; **(D)** evasion of growth suppression; **(E)** genome instability and mutation; **(F)** angiogenesis, **(G)** invasion and metastasis, **(H)** tumor-promoting inflammation, and **(I)** Immune scape. Stromal and immune cells providing the soluble factors driving cancer hallmarks are also highlighted, namely tumor-associated macrophages, dendritic cells, adipocytes, and fibroblasts.

Malignant cells are characterized by uncontrolled proliferation, largely due to the loss of homeostasis in the production, release, and affinity for growth-promoting signals. That said, cancer cells may rely on autocrine proliferative signaling or stimulate stromal cells to supply them with mitotic factors to sustain proliferation. For instance, endothelial and infiltrating immune cells secrete growth-promoting factors that paracrinaly stimulate neoplastic cells proliferation independently from blood-borne factors (42, 43). Moreover, tumor and immune cells-promoted ECM remodeling uncages mitogenic agents while disabling growth suppressing adhesion complexes, thereby maintaining the proliferative potential of cancer cells (44). Furthermore, several ECM proteoglycans, mainly produced by cancer-associated fibroblast (CAF), regulate proliferative signaling in adjacent tumor cells (Figure 2A). For instance, CAF-derived proteoglycans syndecan-1 and versican promote proliferation of human breast cancer cells (45–47) and myeloma tumors (48), mainly by influencing EGF receptor signaling. Likewise, transmembrane syndecan-2 expression appears to be critical for colon carcinoma cell behavior by mediating increased adhesion and proliferation (49). Also, the ECM multifunctional heparan sulfate proteoglycan perlecan strongly augments the binding and mitogenic activity of basic fibroblast growth factor (bFGF), contributing to sustained tumor cell proliferation by FGF pathway activation (50). In line with this, fibroblast-derived hyaluronic acid (HA) paracrinally enhances the *in vitro* proliferation of melanoma cells, while proteins secreted by tumor cells further increase HA synthesis in CAFs in a phosphatidylinositol 3/mitogen-activated protein-kinase-dependent manner (51). On the other hand, the small leucine-rich proteoglycan decorin, expressed primarily by myofibroblast, autocrinally, and paracrinally reduces tumor growth and metastasis in murine xenograft models by downregulating EGFR and Met receptors (52), while inhibiting tumor growth factor β (TGF-β) signaling (53). Decorin also activates ERBB4, which blocks the phosphorylation of heterodimers containing either ERBB2 or ERBB3, thereby suppressing cell growth in mammary carcinoma cells (54). These findings suggest that CAF-derived proteoglycans mainly act as positive regulators of sustained proliferative signaling. In line with this, adipocyte-derived ECM collagen VI affects early mammary tumor progression *in vivo* via signaling through the NG2/chondroitin sulfate proteoglycan receptor expressed on tumor cells (55). Thereby, stromal adipocytes also constitute active players in driving tumor cell proliferation. Of note, the mechanisms through which proteoglycans enforce their action are not fully elucidated and the true implications of GAG chains are yet to be fully clarified. Given these insights, the reciprocal communication between neoplastic and stromal cells is essential to maintain mitogenic factors supply to sustain cellular proliferation.

Glycosylation adds a second level of proliferation regulation by mediating growth factor receptor activation and structural alterations (Figure 2A). Namely, the *O*-GlcNAc modification of transcription factors involved in cell cycle progression, such as factor forkhead protein M1 (FoxM1), cyclin D1, and c-MYC, stabilizes them and contributes to oncogenesis (56, 57) (Figure 2A). Moreover, numerous cell-surface tyrosine kinase receptors (RTK), including EGFR, FGFR, PDGF, c-MET, ERBB2/HER2, and IGFR are known to be regulated by cancer-associated glycans (58–60), glycosyltransferases (61), and proteoglycans (62–65). For instance, the degree of *N*-glycan branching of several RTKs contributes to its capability to induce or arrest cellular proliferation (66, 67). Showcasing this, studies with CHO cells demonstrated that the Asn418-linked *N*-glycan in ERBB3 plays an essential role in regulating receptor heterodimerization with ERBB2 (59), providing a pivotal checkpoint where *N*-glycans may regulate key cellular processes involved in cell proliferation and transformation.

Moreover, core 1 β1,3-galactosyltransferase (C1GALT1, responsible for Tn antigen biosynthesis) overexpression in hepatocellular carcinoma activates hepatocyte growth factor (HGF) signaling via modulation of MET kinase *O*-glycosylation and dimerization, thereby enhancing cell proliferation *in vivo* and *in vitro* (61). Contrastingly, overexpression of β1,4-*N*-acetylglucosaminyltransferase III (MGAT3), which adds β1,4 bisecting branches to *N*-glycans, appears to inhibit EGFR sensitivity to EGF in glioma cells (58), thereby reducing cellular response to the proliferative effects of EGF. In turn, β1,6-*N*-acetylglucosaminyltransferase V (MGAT5) knockout mice were shown less prone to mammary tumor growth and metastasis, while showing poor PI3K/AKT activation, emphasizing the importance of β1,6-GlcNAc-branched *N*-glycans in proliferative signaling pathways (68). Also, ABO glycosyltransferase mRNA downregulation in normal and malignant urothelium is associated with EGF stimulation, resulting in decreased cell proliferation (69). Together, these findings highlight the relevance of glycosyltransferases in tumor cell proliferative signaling.

In turn, the short-chain *O*-GalNAc STn antigen is mainly observed in non-proliferative tumor areas of highly proliferative bladder tumors (70), while being overexpressed in less proliferative hypoxic bladder cancer models (29), suggesting a yet unknown indirect regulation of proliferation by *O*-glycosylation in bladder cancer. In ovarian cancer cells, the knockout of core 1 synthase chaperone *Cosmc*, resulting in Tn and STn *O*-glycans expression, leads to a reduction in cellular proliferation compared to the parental cell lines (71). Moreover, the use of *O*-glycan inhibitors in colorectal cancer cell lines promptly blocks proliferation in a so far unexplored manner (72). Overall, short-chain *O*-glycans expression seem to reduce tumor cell growth. This process might actually confer selective advantage to tumor cells which are rendered less responsive to conventional chemotherapy that mostly targets highly proliferative clones (73).

In addition to alterations in core *O*- or *N*-glycans, changes in terminal glycan structures may likewise induce changes in cell proliferation. For instance, in aggressive non-small cell lung cancer cell lines, knockdown of α1,6-fucosyltransferase 8 (*FUT8*), catalyzing the addition of fucose in alpha 1-6 linkage to GlcNAc residues, significantly inhibits cell proliferation (74). Moreover, overexpression of sialyltransferases and α1,3-fucosyltransferases (*FUT4* or *FUT6*) would suppress EGFR dimerization and phosphorylation upon EGF treatment, decreasing lung cancer cells proliferation (60). In line with this, enhanced α2–6 sialylation, secondary to overexpression of ganglioside-specific *ST6GalNAcV*, inhibits glioma growth *in vivo* (75, 76). Altogether, these findings demonstrate the pleiotropic and occasionally opposing effects of altered glycosylation in cell proliferation.

In summary, these examples demonstrate how the microenvironment and glycosylation can sustain proliferative signals. Overall, the crosstalk between neoplastic cells and the TME ensures the positive feedback look of growth factors supply and ECM remodeling, while glycosylation promotes the exposure and interaction of protein domains with RTKs as well as the constitutive activation of oncogenic pathways through kinases modification.

### Resistance to Cell Death

The TME aids programmed cell death evasion by providing survival signals and offering a physical barrier against pro-apoptotic factors such as chemotherapy. First, endothelial cells establish vasculature to attenuate cell death that would otherwise result from hypoxia and lack of serum-derived nutrients (77). However, when neovascularization cannot keep up with nutrient demand, an hypoxic microenvironment is established where HIF-1α drives antiapoptotic changes (78). In addition, infiltrating macrophages circumvent apoptosis of cancer cells by shielding them from external apoptotic factors and chemotherapy (79). Similarly, CAFs are highly implicated in apoptotic signaling evasion by secreting paracrine survival factors and inducing ECM remodeling (80–82). Moreover, CAF-derived chondroitin sulfate proteoglycan serglycin (SRGN) induces lung cancer chemoresistance and anoikis-resistance, promoting malignant phenotypes through interaction with tumor cell receptor CD44 (83). In addition, ECM proteoglycans as the small leucine-rich lumican promote melanoma cells apoptosis, ultimately inhibiting metastasis to the lungs (84). Consistent with the changes in ECM composition and topography, expression of many ECM remodeling enzymes is often deregulated in human cancers as tumor cells acquire anchorage independence for survival (85). In this context, tumor cell-ECM interactions control malignant cells subversion of positional information and basement membrane dependence to evade apoptosis upon ECM detachment during cancer progression (86, 87). Furthermore, the ECM also aids tumor cells chemotherapy-induced apoptosis evasion (88–90). Likewise, cancer-associated adipocytes are an abundant source of pro-survival factors and extracellular matrix components, specially collagen VI which confers resistance to cisplatin-induced death in ovarian cancer cells (90, 91).

Glycosylation mostly influences the extrinsic apoptotic program, involving both TRAILR and Fas death receptors, as well as integrin and galectin-mediated signaling (Figure 2B). Several glycans, glycosyltransferases, and glycosidases play critical roles in programmed cell death (92) by hindering ligand–receptor interactions, which influences the formation of signaling complexes, and modulating ligand secretion from effector cells (92, 93). For instance, the tumor necrosis factor–related apoptosis-inducing ligand (Apo2L/TRAIL) promotes tumor cell apoptosis through the death receptors TRAIL-R1 and TRAIL-R2, whose *O*-glycosylation status determines its sensitivity to the ligand. Specifically, the *O*-glycosylation initiating enzyme GALNT14 showed a strong link to TRAIL sensitivity in pancreatic carcinoma, NSCLC and melanoma, whereas expression of GALNT3, along with the *O*-glycan processing enzymes FUT3 and FUT6, correlated with responsiveness in colorectal cancer cells, rendering helpful data for identifying cancer patients who are more likely to respond to TRAIL-based therapies (93). Consistent with these observations, a lower degree of fucosylation, which occurs by mutation of the GDP-mannose-4-6-dehydratase (*GMDS*) gene, increases resistance to TRAIL-induced apoptosis in colon cancer cells, followed by immune escape (94). Moreover, *N*-glycosylation also plays an important regulatory role in TRAIL-R1-mediated apoptosis, but not for TRAIL-R2, which is devoid of *N*-glycans. In this context, defective apoptotic signaling by *N*-glycan-deficient TRAIL receptors was associated with lower TRAIL receptor aggregation and reduced death-inducing signaling complex (DISC) formation, but not with reduced TRAIL-binding affinity (95).

In turn, the death receptor Fas (CD95/APO-1) has two *N*-glycosylation sites at N136 and N118 moderately affecting Fas-induced apoptosis. Specifically, the addition of sialic acids by ST6Gal-I in an α2-6 linkage to the *N*-glycans of Fas provides protection against Fas-mediated apoptosis in colon carcinoma cells. Namely, α2-6 sialylation of Fas prevents FasL-induced apoptosis by decreased activation of caspases 8 and 3, blockage of Fas–Fas-associated death domain (FADD) association with Fas cytoplasmic tails, and inhibition of Fas internalization (96). In line with this, high-grade tumors, which are known to express high levels of *O*-6 sialylation, significantly overexpress Fas, but are insensitive to Fas-ligand, thereby avoiding immune cell-mediated apoptosis (30, 97, 98). Moreover, *N*-deglycosylation of Fas leads to the slowing down of procaspase-8 activation at the DISC complex, with no impact on DISC formation or FADD recruitment (99). Overall, these findings demonstrate that, in contrast to the TRAIL-R *O*-linked glycan moiety, the Fas *N*-glycan structure contributes to a smaller extent to the initiation of the apoptotic signaling leading to cell death.

Glycosyltransferases, as *N*-acetylgalactosaminyltransferase 1 (GALNT1), also contribute to activate survival signals that supress apoptosis. Specifically, overexpression of *N*-acetylgalactosaminyltransferase 1 (*GALNT1*) contributes to aberrant glycosylation of integrin α3β1, changing the conformation of integrin heterodimers, and initiating signal transduction to induce focal adhesion kinase (FAK) activation in bladder cancer cells (100). Accordingly, both the knockdown of FAK and suppression of FAK phosphorylation were able to induce apoptosis in BC cells through caspase-3 recruitment and Src phosphorylation, respectively (101). The suppression of FAK phosphorylation also inhibited the PI3K/AKT signaling pathway, suggesting it acts downstream of FAK to regulate apoptosis (101). Interestingly, FAK is overexpressed in a variety of human tumors where it mediates survival signaling, and these findings might point an intervention strategy to regulate apoptotic stimuli through glycosyltransferases modulation.

In addition, several studies suggest that hyper-*O*-GlcNAcylation in cancer may play an anti-apoptotic role (Figure 2B). For instance, human pancreatic ductal adenocarcinoma cells are supported by oncogenic NF-κB transcriptional activity and both NF-κB p65 subunit and upstream kinases IKKα/IKKβ are *O*-GlcNAcylated. As such, reducing hyper-*O*-GlcNAcylation decreases NF-κB transcriptional activity and target gene expression, driving apoptosis (102). Furthermore, increasing *O*-GlcNAc in pancreatic cancer cells protects against suspension-induced apoptosis (102). Moreover, hyper-*O*-GlcNAcylation could contribute to cancer cell survival by mitigating ER stress through the inhibition of the folding enzyme chaperone CHOP (103).

Another important molecular mechanism relating protein glycosylation to apoptosis in cancer cells results from the crosstalk between lectins and death receptors. Classically, the effect of Galectin-3 (Gal-3) in the regulation of apoptosis depends on its subcellular localization. Accordingly, cytoplasmic Gal-3 is anti-apoptotic, whereas nuclear Gal-3 is pro-apoptotic (104). Upon extracellular secretion via a non-classical pathway (105), Gal-3 may bind to cell surface glycans, increasing cell signaling and cell-matrix interactions (106, 107). Interestingly, overexpression of STn results in decreased Gal-3 at the cell surface in colon cancer cells, promoting an accumulation of Gal-3 in the cytoplasm and reducing chemotherapy induced apoptosis (108). Moreover, it has been shown that *O*-6-sialylation of integrin β1 *N*-glycans, mediated by ST6Gal-I, completely blocked its recognition by Gal-3; conversely *O*-3-sialylation did not affect Gal-3 recognition in gastric cancer (108, 109). These observations suggest that Gal-3 binding to glycans is dependent on sialylation and that decoding the sialome of cancer cells may bring new insights on programmed cell death pathways.

Together, these findings demonstrate that both glycosidic and microenvironmental cues aid tumor cells to circumvent apoptosis. Interestingly, the tumor microenvironment mostly provides factors to evade intrinsic apoptotic signaling, while glycosylation mostly regulates the extrinsic signaling pathway initiated by binding of a death ligand to a death receptor on the cell surface.

### Deregulated Cellular Energetics

The microenvironmental modulation of tumor cell energetics is crucial to drive metabolic adaptation and survival of neoplastic cells. As such, CAFs and endothelial cells are able to create collaborative metabolic domains by activating complementary metabolic pathways to buffer and recycle metabolites of tumor cells in order to maintain stromal and tumoral growth (110, 111). Adipocytes also engage in this metabolic crosstalk by providing fatty acids utilized by cancer cells to generate ATP via mitochondrial β-oxidation in metastatic ovarian cancer (112). Another pivotal microenvironmental feature driving energetic adaptation is hypoxia, resulting from uncontrolled proliferation and inefficient neovascularization. Hypoxic stress within a tumor leads to a shift from aerobic oxidative phosphorylation to anaerobic glycolysis, with high rates of glucose and glutamine uptake (the Warburg effect) (113). In this context, adaptation to hypoxia and cellular energetic reprograming occurs mostly in a HIF-1α-dependent manner, being frequently accompanied by cell dedifferentiation and acquisition of mesenchymal characteristics (29). Briefly, to compensate the reduction of intracellular ATP levels under hypoxic conditions, HIF-1α upregulates the expression of glucose transporters-1 and 3 (GLUT1, GLUT3), allowing the intracellular uptake and phosphorylation of glucose (114–116). Subsequently, Glc-6-P enters one of several possible biosynthetic pathways, namely glycolysis, hexosamine biosynthetic pathway (HBP), pentose phosphate pathway (PPP), or glycogen synthesis, all of which substantially regulated by HIF-1α (117–124) (Figure 2C). Simultaneously, HIF-1α decreases O_2_ consumption and reactive oxygen species (ROS) generation within the mitochondria (125–127) to circumvent oxidative stress.

By regulating the flux through the HBP and PPP pathways, HIF-1α dramatically affects glycosylation, either by altering precursor production or by governing enzymatic activity. Specifically, HIF-1α has significant impact on HBP by inhibiting the TCA cycle and suppressing the addition of acetyl groups, that would otherwise arise from that pathway, to glucosamine, leading to an overall reduction in the glycosylation precursor UDP-*N*-Acetylglucosamine (UDP-GlcNAc) production (128–130). Another branch of the HBP, the CMP-NeuAc nucleotide sugar biosynthesis pathway, is activated under hypoxia through the epimerization of UDP-GlcNAc by UDP-GlcNAc 2-epimerase (GNE), ultimately enabling cell surface sialylation in a HIF-1α-dependent manner (131) (Figure 2C).

Moreover, during acute hypoxia, the production of ATP, GTP, UTP, and CTP nucleotides through the PPP is decreased, compromising the addition of UDP to GlcNAc (132). Interestingly, while hypoxia causes downregulation of the rate limiting enzyme of the PPP Glucose-6-phosphate dehydrogenase (G6PD) in several cancers (133), glycosylation promotes G6PD activity and increases glucose flux through the PPP, providing precursors for nucleotide and lipid biosynthesis, and reducing equivalents for antioxidant defense. Particularly, G6PD is dynamically *O*-GlcNAcylated in response to hypoxia, and blocking G6PD glycosylation reduces cancer cell proliferation *in vitro* and *in vivo* (134), most likely through energetic unbalance. On the same note, blockage of hypoxia induced *O*-GlcNAcylation at serine 529 of phosphofructokinase 1 (PFK1) reduced cancer cell proliferation *in vitro* and impaired tumor formation *in vivo* (135). Of note, it has been reported that elevated *O*-GlcNAcylation in cancer cells stabilizes HIF-1α in an indirect manner, thereby reinforcing the Warburg effect (103) in what appears to be negative feedback loop toward homeostatic *O*-GlcNAcylation levels.

In addition to intracellular glucose metabolism modifications, decreased 1,2-fucosylation of cell-surface glycans, galectin overexpression, and glycosyltransferases as well as glycosidases modulation toward the expression of short-chain sialylated *O*-glycans are some consequences of the hypoxic tumor microenvironment. Additionally, increased expression of gangliosides carrying *N*-glycolyl sialic acids can also be significantly affected by hypoxia (29, 136). For all these reasons, it is possible to realize that hypoxia strongly alters glycobiologic events within tumors, resulting in increased *O*-GlcNAcylation and sialylation; thereby leading to more aggressive phenotypes (136–138).

Besides regulating glycolytic enzymes in the context of hypoxia, *O*-GlcNAcylation also governs transcription factors activity (ChREBP, carbohydrate-responsive element-binding protein, Sp, and c-MYC) toward increased aerobic glycolysis, anaplerotic resupply of TCA intermediates used in biosynthesis, nucleotide metabolism and lipogenesis (139–144). Together, these findings suggest that hyper-*O*-GlcNAcylation contributes to oncogenicity through metabolic reprograming and stabilization of oncogenic transcription factors.

Based on these insights, hypoxia is a major driving force of the energetic reprograming of cancer cells, largely affecting glycosylation in a HIF-1α-dependent manner. As such, both *O*-GlcNAc modifications and HIF-1α transcriptional activity emerge as key metabolic modulators, while stromal cells promote a metabolic symbiosis with tumor cells envisaging tumor survival and growth.

### Evasion of Growth Suppressors

To prevail, cancer cells not only induce and maintain stimulatory growth signals but also develop the ability to evade the negative regulation of tumor suppressor genes (145). Even though tumor growth suppression is mostly regulated by intrinsic mechanisms involving p53 and retinoblastoma (RB) pathways, some stromal and microenvironmental components have been implicated in growth arrest evasion by inhibiting adhesion complexes and promoting clonal selection. Namely, proteolytic enzymes produced by stromal cells are able to disrupt cell-cell or cell-ECM adhesion complexes significantly contributing to uncontrolled cell proliferation and progressive distortion of normal tissue architecture (85, 146, 147). Moreover, tumor hypoxia selects clones expressing mutant p53, facilitating the clonal expansion of cells that have a dominant-negative effect on the wild-type cells, thus evading growth suppression (148).

Interestingly, the two canonical suppressors of cell proliferation, p53 and RB, are regulated by *O*-GlcNAcylation (149, 150) (Figure 2D). Particularly, it was demonstrated that p53 *O*-GlcNAcylation on Ser149 limits both ubiquitin-dependent proteasome degradation and the interaction with E3 ubiquitin-protein ligase MDM2 (149). Contrariwise, overexpression of *O*-GlcNAcase (OGA) results in increased MDM2 phosphorylation at Ser166, stimulating MDM2-p300 interactions and resulting in p53 degradation (151). In turn, RB activity is regulated by the dynamic crosstalk between *O*-GlcNAc modification and phosphorylation (150). Retinoblastoma binds E2F-1 transcription factor preventing co-activator complexes from binding E2F-1, thereby arresting cell cycle in the G1 phase. Particularly, RB is densely modified with *O*-GlcNAc in the G1 phase, which prevents its phosphorylation and sustains its activity. During mid- to late-G1, a shift toward increased phosphorylation leads to the release of E2F-1 from RB and E2F-1-dependent transcriptional activation of essential S-phase genes, allowing cell cycle progression (150).

In summary, cancer cells circumvent growth suppression by negatively regulating the two canonical suppressors of proliferation p53 and RB through glycosidic modifications, while stromal cells and hypoxia aid tumor cell growth by abrogating the suppressive role of adhesion complexes and selecting for more proliferative clones.

### Genome Instability and Mutations

During uncontrolled cell division, random mutations, and chromosomal instability promote genomic alterations, which coupled with disruption of genome integrity checkpoints culminate in selective advantage of tumor cells (152). In this context, intratumoral hypoxia leads to increased mutation rates and altered DNA damage response, while HIF-1α interplays with oncoproteins such as c-MYC to drive malignant progression (153–155). In addition, recent evidence shows that oxidative stress in CAFs induces genomic instability in adjacent breast cancer cells via mutagenic evolution, potentially increasing their aggressive behavior (156). Together, these findings suggest that tumor progression is prompted by the orchestrated interaction of malignant cells and the TME, which promotes genetic instability toward more aggressive phenotypes.

It is known that the tumor suppressor p53 plays a central role in genomic stability maintenance (157). However, stabilization of previously mutated p53 by *O*-GlcNAcylation is not expected to lead to tumor suppression (149). Nevertheless, SILAC-based quantitative proteomics of *O*-GlcNAc transferase wild-type and Null cells has demonstrated the *O*-GlcNAcylation regulation of the ATM (ataxia-telangiectasia mutated)-mediated DNA damage response pathway through ATM and its downstream targets H2AX, and Chk2 (158) (Figure 2E). Other molecular studies have reinforced that ATM interacts with *O*-GlcNAc transferase, with its activation and recovery states being affected by *O*-GlcNAcylation (159).

Importantly, genetics is not the only factor contributing to genetic instability. Epigenetic modifications through DNA methylation, posttranslational modification of histone proteins, and interactions of non-coding RNAs with proteins or other nucleic acids also largely drive cancer progression (160, 161). Interestingly, histones H2A, H2B, and H4 are *O*-GlcNAcylated *in vivo*, making *O*-GlcNAc modifications a part of the histone code regulating gene transcription (162). Although no specific links between hyper-*O*-GlcNAcylation and cancer cell epigenetic contribution to transformation have been established, some clonal expansions may well be triggered by these non-mutational changes affecting the regulation of gene expression.

In summary, tumor microenvironmental features and stromal cells contribute to a mutagenic environment through the production of oxygen and nitrogen reactive species, while altering transcription and translation of several DNA damage response and repair genes. In turn, glycosylation modulates DNA damage response pathway components and possibly non-mutational changes affecting the regulation of gene expression.

### Replicative Immortality

The maintenance of telomerase lengths by DNA polymerase telomerase is a key event contributing to the unlimited replicative potential of cancer cells (163). Recently, hotspot point mutations in the regulatory region of the *telomerase reverse transcriptase (TERT)* gene, encoding the core catalytic component of telomerase, was identified as a novel mechanism to activate telomerase in cancer (164, 165). Interestingly, there is currently no substantive evidence of microenvironmental contributions to telomere stabilization in cancer cells. However, there is evidence that hypoxia up-regulates telomerase activity in cancer cells via MAPK cascade signaling activation as a stress response against hypoxia-induced genotoxicity (166). Moreover, hypoxia induces c-MYC activation, which, in turn, transactivates *TERT* (167). So far, TERT has not been described as a glycoprotein; nevertheless, there could be an indirect link between glycosylation and telomerase activation through c-MYC *O*-GlcNAcylation regulation (57). As such, future studies should investigate whether *O*-GlcNAc-mediated stabilization of c-MYC can indirectly influence telomerase activation and contribute to replicative immortality.

In conclusion, both glycosylation and microenvironmental factors allow successive cell cycles mostly by circumventing cell death, while having little to do with avoiding senescence and regulating telomere length. However, tumor hypoxia might contribute to immortalization by indirectly influencing kinase cascades and transcriptions factors, while glycosylation modifications have a more modest impact in transcription factor regulation.

### Angiogenesis

The formation of neovasculature through angiogenic processes is vital for cancer cell proliferation and tumor progression to metastasis (168). Historically, tumor angiogenesis was perceived as being primarily regulated by cancer cells expressing proangiogenic factors; however, now it becomes increasingly clear that the tumor microenvironment is a key factor inducing and sustaining chronic angiogenesis, including in a glycosylation-dependent manner. First, tumor hypoxia upregulates multiple pro-angiogenic pathways mediating key aspects of stromal, endothelial cell (EC) and vascular support cell biology to influence neovessel patterning, maturation, and function (169). Concomitantly, stromal innate immune cells and CAFs synthesize or release through ECM remodeling several angiogenic soluble factors driving the expansion of the pre-existing vascular supply (170–174). In line with this, stromal cells-derived proteoglycans and ECM molecules are also active angiogenesis regulators (Figure 2F). For instance, heparan sulfate (HS) proteoglycans inhibition hampers pro-angiogenic signaling and neovessel formation by effecting the bioactivity, diffusion, half-life and interaction of VEGF with its tyrosine kinase receptors (175, 176). In ovarian cancer, HS has also been shown to impact angiogenesis through EGF receptor signaling and by influencing the expression of angiogenic cytokines (177). Particularly, CAF-derived HS proteoglycan syndecan-1 expression stimulates breast tumor angiogenesis, being correlated with both vessel density and total vessel area (178). Furthermore, Neuropilin-1 (NRP-1) and Neuropilin-2 (NRP-2) transmembrane proteoglycans, as well as hyaluronic acid (HA) fragments resulting from the hydrolysis of carbohydrate chains in proteoglycans by HYAL hyaluronidase, also display pro-angiogenic properties in several cancer models (179–182). Contrastingly, stromal decorin angiogenic role seems to be context dependent. Namely, it blocks tumor cell-mediated angiogenesis by downregulating VEGFA production, as well as Met and downstream angiogenic networks in some tumor models (183, 184), while being required for efficient tube formation by EC and inflammation-induced angiogenesis in others (185). In turn, the basal lamina lumican, a class II small leucine-rich proteoglycan, inhibits melanoma angiogenesis by compromising the migratory capacity of EC and pseudotubes formation, supressing lung metastasis (84). Moreover, lumican affects angiogenesis by interfering with α2β1 integrin receptor activity and downregulating proteolytic activity associated with surface membranes of EC (186). In line with this, several studies highlight that lumican inhibits EC invasion, angiogenic sprouting, and vessel formation, while enhancing Fas mediated EC apoptosis (187–190). Collectively, these findings provide new insights into how ECM remodeling regulates angiogenesis activation and resolution, as well as identify proteoglycans as effectors modulating angiogenesis both *in vitro* and *in vivo*.

Glycans and glycan-binding proteins, as galectins, add another level of positive regulation of angiogenesis by modulating EC migration, branching, survival, and vascular permeability (191–193). For instance, a glycosylation-dependent pathway that preserves angiogenesis in response to VEGF blockade was identified, in which galectin-1 (Gal-1) binds β1-6GlcNAc branched *N*-glycans present on VEGFR2 in EC surface to activate a VEGF-like signaling (Figure 2F). Moreover, vessels within anti-VEGF-sensitive tumors exhibited high levels of α2-6-linked sialic acids, which prevented Gal-1 binding and VEGFR2 activation (192). Moreover, interruption of β1-6GlcNAc branching in EC or silencing of tumor-derived Gal-1 converted refractory tumors into anti-VEGF-sensitive (192). Importantly, this could allow pinpointing patients better served by anti-VEGF therapy and targeting glycosylation-dependent lectin-receptor interactions envisaging increased treatment efficacy in refractory patients (194, 195).

In addition, reduced *O*-GlcNAcylation in prostate cancer cells has been associated with decreased expression of several angiogenic factors, such as matrix metalloproteinases MMP-2 and MMP-9, and VEGF, resulting in inhibition of angiogenesis (196). Moreover, glycosydic cues as *O*-glucose, *O*-GlcNAc, and *O*-GalNAc glycans affect Notch signaling, thereby regulating angiogenesis (197). Also, α2,6-sialic acids are necessary for the cell-surface residency of platelet endothelial cell adhesion molecule (PECAM), a member of the immunoglobulin superfamily that plays multiple roles in EC adhesion, mechanical stress sensing, anti-apoptosis, and EC-mediated angiogenesis (198). Together these finding highlight the glycosylation modulation of tumor angiogenesis.

In summary, the tumor microenvironment ensures the supply of pro-angiogenic factors, while upregulating multiple pro-angiogenic pathways governing the maturation and survival of endothelial cells. In turn, glycans and glycoconjugates can be angiogenic *per se* or alter the affinity of angiogenic factor receptors for their ligands toward a pro-angiogenic phenotype of EC.

### Invasion and Metastasis

Throughout the course of disease, cancer cells often acquire more motile phenotypes, as well as the capability to invade surrounding tissues and adjacent organs. Subsequently, cancer cells reach lymph and blood vessels, entering circulation and eventually metastasizing to distant locations. Interestingly, metastatic tumor cells may even travel from the primary site to the secondary location with stromal components, including activated fibroblasts, achieving a very favorable outcome in the colonization step of tumor progression (199). In this context, stroma, ECM, and microenvironmental cues often facilitate invasion and the establishment of metastatic colonies by tumor cells. For instance, tumor hypoxia aids migration and invasion of tumor cells by influencing angiogenesis, immune tolerance, epithelial-to-mesenchymal transition (EMT), and regulating adhesion molecules expression and glycosylation (200). At a distance, hypoxia contributes to the production of diffusible factors and exosomes involved in premetastatic niche formation, while regulating metabolic and survival pathways that allow cells to adapt to distant microenvironments (201). Within the tumor stroma, infiltrating immune cells and CAFs promote ECM remodeling while producing pro-invasive and EMT promoting factors (172, 202–204). Namely, the CAF-derived proteoglycans versican and serglycin promote tumor invasion and metastasis in breast, ovarian, and prostate cancer (47, 205, 206), as well as NSCLC cells EMT, migration, invasion and liver colonization, respectively (83). Similarly, the ECM hyaluronic acid (HA) and biglycan are directly involved in the metastatic potential of breast and prostate tumor cells (207, 208) as well as melanoma cells (209), respectively. Moreover, metastatic tumor cells must acquire the capability to autonomously synthesize, assemble, and process their own “portable” HA-rich microenvironments to survive in circulation, metastasize to ectopic sites, and escape therapeutic intervention. As such, strategies to disrupt the HA machinery of primary tumor and circulating tumor cells may enhance the effectiveness of current conventional and targeted therapies (210, 211). On the other hand, triple-negative orthotopic breast carcinoma systemic treatment with the proteoglycan decorin induced the tumor suppressor cell adhesion molecule 1 (*Cadm1*), favoring a less metastatic phenotype (212, 213). Altogether, these findings highlight stromal-derived proteoglycans as major players driving the metastatic potential of tumor cells. Concomitantly, *in vitro* studies suggested that stromal derived TGFβ-induced EMT alters glycogenes expression and consequently promotes *N*-glycan remodeling, including decreased bi-, tri- and tetra-antennary complex *N*-glycans and increased expression of hybrid-type *N*-glycans and fucosylation (214); thereby showing a correlation between microenvironmental soluble factors and glycosylation changes.

In line with glycoconjugate regulation of invasion and metastasis, glycans add another dimension of regulation to the acquisition of this cancer hallmark. Namely, it has been proposed that increased sialylation, accompanying malignant transformation, promotes cell detachment from the primary tumor through electrostatic repulsion of negative charges, physically disrupting cell adhesion (215, 216). In line with this, the STn antigen reduces cell adhesion in prostate cancer (217), while increasing migration and invasion in bladder (29), breast (218), and gastric (219, 220) carcinomas in a *ST6GalNAc.I*-dependent manner. Also, the increased and *de novo* expression of the STn antigen in bladder cancer cells is part of an array of molecular events underlying the establishment of mesenchymal traits (29). Moreover, STn was mainly found in densely *O*-glycosylated adhesion proteins such as integrins and cadherins (29, 30). It is likely that the transition from extended to shorter and heavily sialylated structures may impair these proteins normal function and induce molecular and spatial reorganization at the cell-cell and cell-matrix interfaces. In agreement with these observations, STn expressing cells are frequently simultaneously observed in invasion fronts, near blood vessels and corresponding lymph nodes, as well as in distant metastasis (70, 221). Moreover, it has been recently reported that most circulating tumor cells (CTC) in the blood of metastatic bladder cancer patients present a highly undifferentiated and more aggressive basal phenotype, while overexpressing the STn antigen (221). As such, STn expression seems to confer a competitive advantage to neoplastic bladder cells by enabling not only invasion but also the necessary mechanisms for successful cancer dissemination. Similarly, ST6Gal.I-mediated α2,6-sialylation of breast cancer cells mediates reduced cell-cell adhesion and enhanced invasion capacity (222). Overall, immature truncated *O*-glycophenotype of cancer cells directly induces oncogenic features, including enhanced migration and invasive capacity (223).

Reinforcing the key role played by sialic acids in cell-cell adhesion, sialylated α3β1 integrin, displaying numerous sialylated tetra-antennary complex type glycans, exhibited significantly lower fibronectin-binding capability than its unsialylated counterpart and showed migration ability through fibronectin *in vitro* (224). Apart from integrins, E-cadherin aberrant glycosylation highly affects its function and cellular localization, frequently culminating in epithelial cell invasion in gastric cancer (225, 226). Namely, *N*-acetylglucosaminyltransferase III (GnT-III, MGAT3) and *N*-acetylglucosaminyltransferase V (GnT-V, MGAT5) competitively modify E-cadherin *N*-glycans, adding bisecting GlcNAc structures and β1,6-GlcNAc branches, respectively. Wild-type E-cadherin positively regulates the metastasis suppressor *MGAT3* gene, resulting in increased GnT-III expression and bisecting GlcNAc *N*-glycans addition to the plasma membrane-bound protein (225). Conversely, the addition of β1,6-GlcNAc branches by GnT-V, specially at Asn-554, drives E-cadherin translocation to the cytoplasm, alters cis-dimer formation and molecular assembly, and drives instability of the adherens junctions. Furthermore, preventing Asn-554 *N*-glycosylation, either by a mutation or by silencing GnT-V, resulted in a protective effect on E-cadherin, precluding its functional dysregulation and contributing to tumor suppression (226, 227). Another study demonstrated a novel pathway of GnT-V-mediated metastasis via the addition of β1,6-GlcNAc branches to matriptase, thereby stabilizing it and activating invasion effectors as urokinase-type plasminogen activator and hepatocyte growth factor (HGF) (228). Overall, these findings suggest that aberrant *N*-linked β1,6- GlcNAc branching occurring during oncogenesis can lessen cell-cell adhesion, contributing to increased cellular motility and invasiveness (Figure 2G). However, some glycosydic modifications can promote tumor cell adhesion and still favor tumor progression. For instance, tumor cells also overexpress SLe^a/x^ antigens, which are specific ligands for E- and P-selectins upregulated in activated endothelial cells. Selectins and SLe^a/x^ interactions are key regulators of the metastatic cascade by promoting the recruitment of malignant cells to vessels, rolling of tumor cells on the endothelial surface, and arrest of CTCs in distant locations (229–231) (Figure 2G). Besides the establishment of metastatic colonies, these ligands also mediate tumor growth, invasion, angiogenesis, and inflammation in numerous other tumor types (232–236). In addition, slightly altered forms of these antigens also have important biological features. Namely, the addition of a sulfate group at the sixth position of GlcNAc generates 6-sulfo-sLe^X^, which is considered the physiologic ligand for L-selectin (237) but also E-selectin in bladder cancer (238). Herein, it has a dual role by promoting tumor cell adhesion to vascular endothelial cells, while favoring lymphocyte recruitment to enhance anti-tumor immune responses (238). In agreement with these observations, Le^x^-positive cell lines from invasive bladder tumors with metastatic potential show high levels of alpha1,3-fucosyltransferase VI (FT-VI) and FT-VII, two enzymes involved in SLe^x^ synthesis, and display E-selectin dependent adhesion (232).

Glycosyltransferases may also play a key role in mediating cancer cell metastization. Namely, the sialyltransferase ST6GalNAcII was identified as a novel metastasis suppressor, while ST6GalNAcV and *N*-Acetylgalactosaminyltransferase GalNT9 identify metastatic potential in breast cancer (239–241).

In summary, cancer-associated glycosylation changes and stromal cells aid tumor cell invasion, distant organ colonization, and metastasis by supplying pro-metastatic factors, compromising vasculature integrity and the stromal barrier to tumor cell migration, promoting EMT and by tethering tumor cells to improve colonization at distant sites. Concomitantly, the highly regulated balance between loss of adhesive properties and the ability to anchor at metastatic sites defines the metastatic potential of tumor cells.

### Tumor-Promoting Inflammation

Tumor-associated stromal cells have been found to secrete a variety of pro-inflammatory cytokines, chemokines and matrix-remodeling enzymes favoring the establishment of immune cell infiltrates (242, 243). Particularly, CAFs and mature adipocytes promote sustained inflammation by producing large amounts of pro-inflammatory IL-6, IL-1β, TNF-alpha, and CXCL1 to drive chemoattraction of monocytic immune cells (244), while favoring tumor growth and metastasis (245–250). Another pivotal microenvironmental factor driving cancer-associated inflammation is hypoxia, which is essential for granulocytes and monocytes/macrophages infiltration and activation *in vivo* in a HIF-1α-dependent manner (251).

Glycome alterations also decisively contribute to the establishment and maintenance of tumor-promoting inflammation. Namely, E-, P-, and L-Selectins interactions with SLe^a/x^ not only control the establishment of metastatic cancer cells colonies but also the recruitment of circulating lymphocytes into peripheral lymph nodes and inflamed tissues (238, 252, 253) (Figure 2H). Moreover, several inflammatory mediators are regulated by its glycosylation state. Namely, NF-κB is activated by *O*-GlcNAcylation at Ser350 of its c-Rel subunit (254), while the proinflammatory cytokine Cyclooxygenase-2 (COX-2) turnover depends on Asn570 glycosylation, negatively affecting the efficacy of certain COX-2 inhibitors (255, 256). Furthermore, recent studies have described that non-human *N*-glycolyl-neuraminic acid (Neu5Gc) can be incorporated into cell surface glycans instead of *N*-acetyl-neuraminic acid (Neu5Ac), leading to autoimmune systemic inflammation associated with cancer initiation and progression (257–259).

Importantly, in the same way glycans govern inflammation, the inflammatory tumor microenvironment is also able to induce changes in tumor cells glycosylation. For instance, pancreatic and gastric carcinomas are characterized by an abundant stroma containing several pro-inflammatory cytokines, as IL-1β and IL-6, which regulate the expression of biosynthetic glycosyltransferases to increase the expression sialylated antigens as SLe^a/x^ (260, 261). Furthermore, the extracellular matrix proteoglycan versican has been shown to promote bladder cancer-derived lung metastasis through enhanced tumor cell migration and creation of an inflammatory environment involving macrophages and pro-tumor CCL2/CCR2 signaling axis (262, 263), providing another the involvement of glycoconjugates in macrophage-mediated inflammation.

These findings highlight the relevance of tumor stromal cells, glycans, and glycoconjugates as mediators of tumor-promoting inflammation by providing pro-inflammatory factors and allowing the recruitment of circulating lymphocytes into tumor sites.

### Immune Escape

Several stromal components of the tumor microenvironment aid tumor cell immune scape, either by recruiting immunosuppressive immune cells or by driving the acquisition of tolerogenic phenotypes. In this context, tumor-infiltrating immune cells frequently develop immunosuppressive activities, differentiating into regulatory T cells (Tregs), immature monocytes, and alternatively activated macrophages, mast cells, neutrophils, dendritic cells (DC), and T helper 2 (TH2)-CD4^+^ T cells, all of which producing a multitude of factors aiding tumor growth and survival (264). Specifically, endothelial cells lining the tumor vasculature can suppress T cell activity, target them for destruction, and block them from entering the tumor through the deregulation of adhesion molecules (265). Moreover, the CAF secretome can also shape T cell-dependent antitumor immune responses by negatively affecting DCs, myeloid-derived suppressor cells, TH17, and CD8^+^ T cells functions. Activated fibroblasts can also drive the switch of CD4^+^ T lymphocytes from a TH1 to a TH2 phenotype, while expressing some ligands of immune checkpoint receptors (266). CAF-derived proteoglycans, as decorin, further suppress immunomodulatory genes in triple-negative orthotopic breast carcinoma xenografts, including *Siglec* (Sialic acid binding Ig-like lectin), *Lipg* (IFNγ inducible GTPase), and *Il1b* (Interleukin 1β) (213). These findings suggest that targeting CAFs or their secretome may probably reduce immune effector cell dysfunctions as well as decrease the recruitment of immunosuppressive cells. Other ECM molecules, as HA, are known to determine the trafficking of tumor-associated macrophages (TAM) through tumor stromal areas. In line with this, HA deficiency in tumor stroma impairs not only macrophage trafficking but also tumor angiogenesis and lymphangiogenesis, ultimately compromising immune cells access to tumor sites and aiding immune scape (267). Furthermore, recent studies in myeloma tumors have demonstrated the immunomodulatory roles of the ECM proteoglycan versican proteolytic processing. In this context, the interplay between stromal cells and myeloid cells generates versikine, a novel bioactive damage-associated molecular pattern that may facilitate immune sensing of myeloma tumors and modulate the tolerogenic consequences of intact versican accumulation (268).

As described in previous sections, advanced stage tumors are frequently characterized by profound deregulations in glycosylation pathways, resulting in the presentation of aberrant structures at the cell surface. Importantly, these structures only render cancer cells mildly antigenic and rarely immunogenic (269). This may occur because most cancer-associated structures have an embryonic origin or are mildly expressed in healthy tissues, allowing them to be perceived as “self” by immune system effector cells (270). Furthermore, specialized B lymphocytes producing high-affinity antibodies against these structures might even be eliminated during development (271). However, glycans play a key role in the regulation of various aspects of immune response, ultimately enabling immune suppression by interacting with lectin receptors in immune cells. For instance, fucosylated blood group related Lewis antigens interact with C-type lectin DC-SIGN (dendritic cell-specific ICAM-3-grabbing non-integrin; also known as CD209) on macrophages and DC to upregulate the anti-inflammatory cytokines IL-10 and IL-27. This ultimately induces TH2, T follicular helper (TFH) or Treg cells, highlighting the immune suppressive nature of Lewis antigens (272, 273). Similarly to fucosylation, enhanced tumor sialylation often culminates in immune suppression and anti-inflammatory microenvironments. Accordingly, the presence of sialylated structures on melanoma cells impedes T cell mediated anti-tumor responses while promoting tumor-associated Treg cells and decreased NK cell activity (274) (Figure 2I). Moreover, sialoglycans interact with sialic acid-binding immunoglobulin-like lectins (SIGLECs) to induce an antigen-specific tolerogenic programming, enhancing Treg cells and reducing the generation and propagation of inflammatory T cells (275). For instance, macrophage associated Siglec-15 preferentially binds the STn antigen in myeloid tumor cells, resulting in increased TGF-β secretion into the tumor microenvironment and tumor progression (276). Moreover, in bladder cancer, STn expression has led to impaired DC maturation while significantly reducing the production of Th1-inducing cytokines IL-12 and TNF-α (277) (Figure 2I). Consistent with this tolerogenic profile, T cells primed by DCs pulsed with STn-expressing glycoproteins displayed a FoxP3(high) IFN-γ(low) phenotype and little capacity to trigger protective anti-tumor T cell responses (277). More importantly, blocking STn-MUC1 and CD44 glycoforms partially reverted DC maturation, suggesting that targeting STn-expressing glycoproteins may allow circumventing tumor-induced tolerogenic mechanisms. Similarly, sialylation of the T antigen in MUC1 on breast cancer cells creates the MUC1–ST antigen which engages Singlec-9 on tumor-associated macrophages to initiate inhibitory immune signaling through the activation of the MAPK/ERK pathway (278). In line with this, sialylated ligands of singlec-7 and−9 are expressed on cancer cells of different histological types and interactions between these lectin receptors and its ligands influence NK cell-dependent tumor immunosurveillance (279). Moreover, hypersialylation of tumor ligands for NKG2D receptors, expressed by NK cells, NK1.1+ T cells, γδ T cells, activated CD8^+^αβ T cells and macrophages, is thought to repulse their interaction via highly negative charge repulsions, hampering immune response (280, 281). Tumor-derived sialoglycans also inhibit CD8+ T cell cytotoxicity by interfering with lytic granule trafficking and exocytosis in response to TCR engagement (282). Thus, hypersialylation often observed on tumor cells may ultimately be amongst the mechanisms by which tumors evade immune system recognition (30, 70, 216, 283). Also, C2GnT-expressing bladder tumor cells express heavily core 2 *O*-glycosylated MUC1 which interacts with Gal-3 to attenuate the interaction of tumor cells with NK cells, allowing tumor cells to survive longer in host blood circulation and potentially metastasize (284). Given these insights, sialylated and fucosylated antigens contribute to create an immunosuppressive microenvironment toward tumor cell immune escape. Furthermore, the structure and function of well-known immune checkpoint molecules as PD-L1 can be stabilized by *N*-glycosylation, reducing its proteasomal degradation and consequently enhancing its immunosuppressive activity over T-cells (285). These findings highlight the disseminated role of glucans and glycoconjugates in tumor cell immune scape.

In summary, the tumor microenvironment increasingly becomes more immunosuppressive, resulting in tumor cell survival and metastasis. Concomitantly, tumor cells glycosylation promotes immune scape by being simple and “self”-like, by inducing tolerogenic immune cell phenotypes, and by effectively shielding tumor cells from effector immune cells, culminating in tumor progression.

## Significance of Glycosignatures for Personalized Medicine

**Figure 3:**
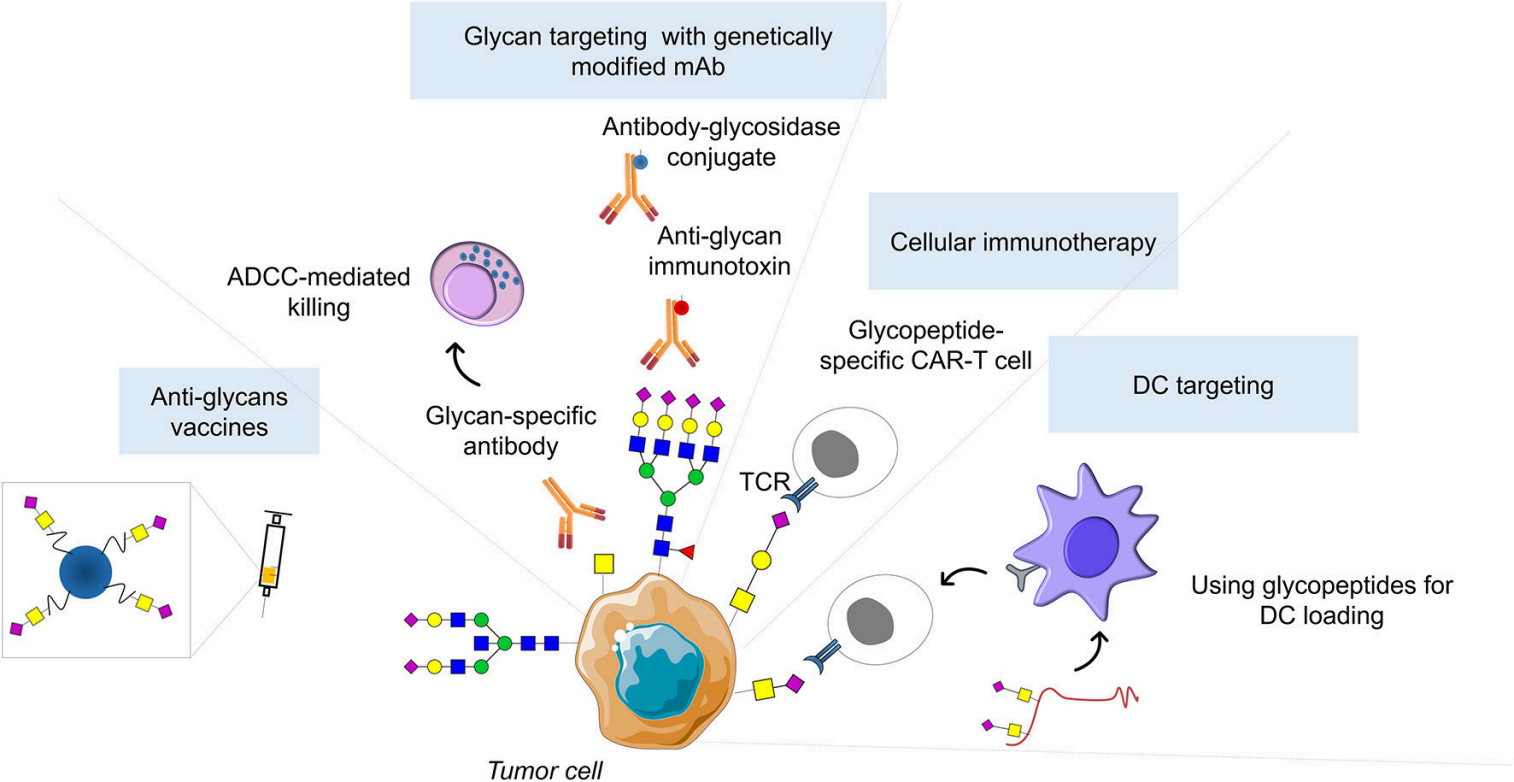
Glycan-based therapeutic strategies. Successful clinical implementation of glycan-based therapeutic strategies could include inhibitors of glycosyltransferases catalytic activity, as well as theragnostic antibodies against cancer glycoepitopes capable of cancer detection, antibody-dependent cellular cytotoxicity induction, and abrogation of immunotolerance generated by cancer-associated glycoconjugates. Moreover, glycan-based antibodies may be used to guide emerging nanotherapies or serve has basis for developing genetically modified T cells expressing chimeric antigen receptors (CART-T). In addition, glycopeptides can be used for *in vivo* targeting of dendritic cells (DCs) to induce tumor-specific CD4^+^ and CD8^+^ T cells. Finally, glycan antigens coupled to T-cell peptide epitopes or immunostimulant epitopes can form fully synthetic multicomponent glycoconjugate vaccines able to circumvent cancer immunotolerance.

The previous sections have highlighted that changes in glycans and glycoconjugates drive several biological processes in tumor cells, culminating in the acquisition of cancer hallmarks and increasingly aggressive disease. Glycosylation changes reflect not only the genomic, transcriptomic, proteomic and metabolomic state of cells but also its external microenvironment, making glycosignatures highly context-specific and attractive targets for personalized medicine affecting tumor and stromal cells. At a systemic level, glycosignatures provide a global reflection on an individual's health/disease status and can function as predictive indicators for treatment success. In this context, several serological markers have emerged, with several FDA-approved cancer glycobiomarkers currently used in clinical practice recently revised by kirwan et al. (286). To circumvent relatively low specificity and sensitivity issues, more comprehensive approaches propose combinations of glycobiomarkers achieving remarkable sensitivity and specificity values (287). Another strategy to improve specificity consists in narrowing the cancer cell proteome to clinically relevant glycoforms. Showcasing this aspect, a recent targeted investigation of the bladder cancer glycoproteome highlighted that specific MUC16 glycoforms (CA125 antigen) could be used to define subsets of chemoresistant patients, whereas no associations could be found based solely on the presence of the protein (30). Moreover, the field of liquid biopsies is rapidly evolving from classical approaches, focusing on a single or few protein biomarkers, toward multiplex settings that will likely improve on these preliminary findings (Figure 3). The detection of minor amounts of circulating tumor nucleic acids, exosomes, circulating tumor cells (CTC) and stromal components, which decisively contribute to the pre-metastatic and metastatic niches, will pave the way for improving the management of advanced stage patients. In this context, deeper insights on their molecular nature may provide the necessary means for real-time disease monitoring and early intervention, guiding therapeutic decision and, more importantly, designing novel therapeutics (Figure 3). Accordingly, explorative studies have demonstrated that exosomes, responsible by pre-metastatic signaling, present distinct glycosylation patterns (288, 289). Furthermore, pioneer work using a recently developed microfluidics device has demonstrated that over 90% of bladder cancer CTC yield the STn antigen (221). More importantly, the STn antigen was not detected in blood cells from healthy individuals, reinforcing its cancer-associated nature. Downstream molecular analysis confirmed the basal nature of STn-positive CTC in molecular mimicry of the primary tumor and corresponding metastasis (221). Therefore, the STn may allow targeting bladder CTC, which has been a challenging enterprise given the scarce knowledge about their molecular nature.

Despite these promising advances, current diagnostic strategies are based on measuring protein marker concentrations, disregarding its glycosylation status, even though it might provide key information to improve diagnosis and stratify patients. This might be due to the lack of user-friendly tools allowing health care technicians to obtain this information in sufficient specificity and sensitivity within the standard capacities of a clinical laboratory. Moreover, the glyco-heterogeneity of protein markers, arising from multiple glycosylation sites and glycosylation patterns, might further hamper selectivity. As such, the profound knowledge of cancer-specific glycan signatures and glycosites, as well as its status within a healthy population represent the first crucial steps toward including glycosylation in the diagnostic process. From the bench side, current glycobiology rationale is mostly built on immunoaffinity-based studies addressing conventionally accepted glycan-biomarkers and involving small and often biased patient cohorts. Heterogeneous protocols, including different sample processing and detection methods, as well as the lack of endpoint standardization have also constituted major drawbacks. Moreover, most studies fail to provide complementary functional assays capable of pinpointing clinically relevant glycobiomarkers. These aspects are often further aggravated by the lack of untargeted approaches capable of broadening our understanding on the glycome and glycoproteome. Moreover, few efforts were undertaken to incorporate glycans in broad biomarker panels of different molecular natures, envisaging highly sensitive and specific detection methods. Facing these challenges, significant efforts are ongoing to standardize glycomics and glycoproteomics protocols and implement robust high-throughput mass spectrometry-based glycoanalytical platforms (290, 291). As such, it is now possible to extract significant structural information from minute amounts of clinical samples (nanomolar-fentomolar range), including from challenging starting materials such as formalin-fixed paraffin-embedded (FFPE) tissues available in many hospital archives (30, 292), which will enable large scale retrospective analysis of well characterized clinical samples. Moreover, advances in MALDI Imaging Mass Spectrometry has allowed obtaining structural information from glycans with significant spatial resolution (293). Important bioinformatics tools and databases are already available and novel improvements are emerging for supporting glycans and glycopeptide mass spectrometry data interpretation, which is a critical matter facing big datasets (294). Altogether, the technological set-up and structural knowledge envisaging the engagement in multicenter randomized glycan-based trials have been overcome; nevertheless, a more ambitious focus should be set on integrative panomics applications (295). This knowledge will foster the development of glycan-based therapeutic strategies and novel immunotherapeutics, including inhibitors of glycosyltransferases catalytic activity (296) and theragnostic antibodies against cancer-specific glycoepitopes. The later should be capable of inducing antibody-dependent cellular cytotoxicity and/or overcoming the immunotolerance generated by cancer-associated glycoconjugates and microenvironmental cues (297). Moreover, glycan-based antibodies may be used to guide emerging nanotherapies (298, 299) or serve has basis for developing genetically modified T cells expressing chimeric antigen receptors (CAR-T) (300), while allowing cancer detection and identification of patients better-served by these therapies. In addition, blocking tumor-associated glycan–lectin interactions could prevent the activation of inhibitory immune receptors toward more efficient immunotherapies. Regarding personalized immunotherapies, in recent years, the targeting of DCs has emerged as an interesting approach for the induction of antitumor immunity. Namely, glycopeptides targeting DC-SIGN in DCs are easily internalized and cross-presented to stimulate tumor-specific CD4^+^ and CD8^+^ T cell responses. Finally, anticancer multicomponent glycoconjugate vaccines, based on glycan antigens coupled to T-cell peptide epitopes or immunostimulant epitopes, have been demonstrated effective in circumventing cancer immunotolerance (301, 302), providing an appealing option for the much-awaited development of new glycan-based therapeutic agents.

In summary, analytical hurdles related with sample preparation, data acquisition and automated analysis that can also be handled by non-glycobiologists represent key steps to overcome to introduce glycomics and glycoproteomics as routine clinical parameters. To achieve this goal, the development of new and clinic-friendly techniques, as well as glycobiology-focused bioinformatics tools open new avenues to predict the tumor glyco-code. In addition, stratification and large-scale validation of potential diagnostic targets will also be indispensable to successfully translate promising research results into solid clinical tests. In a distant future, an inclusive approach combining the increasing amount of glycomics and glycoproteomics data with patient's genomics, transcriptomics, proteomics, and metabolomics will have a major impact on the unraveling of novel targets and strategies for early diagnosis, prognosis, patient stratification and improved cancer management.

## Concluding Remarks

**Figure 4:**
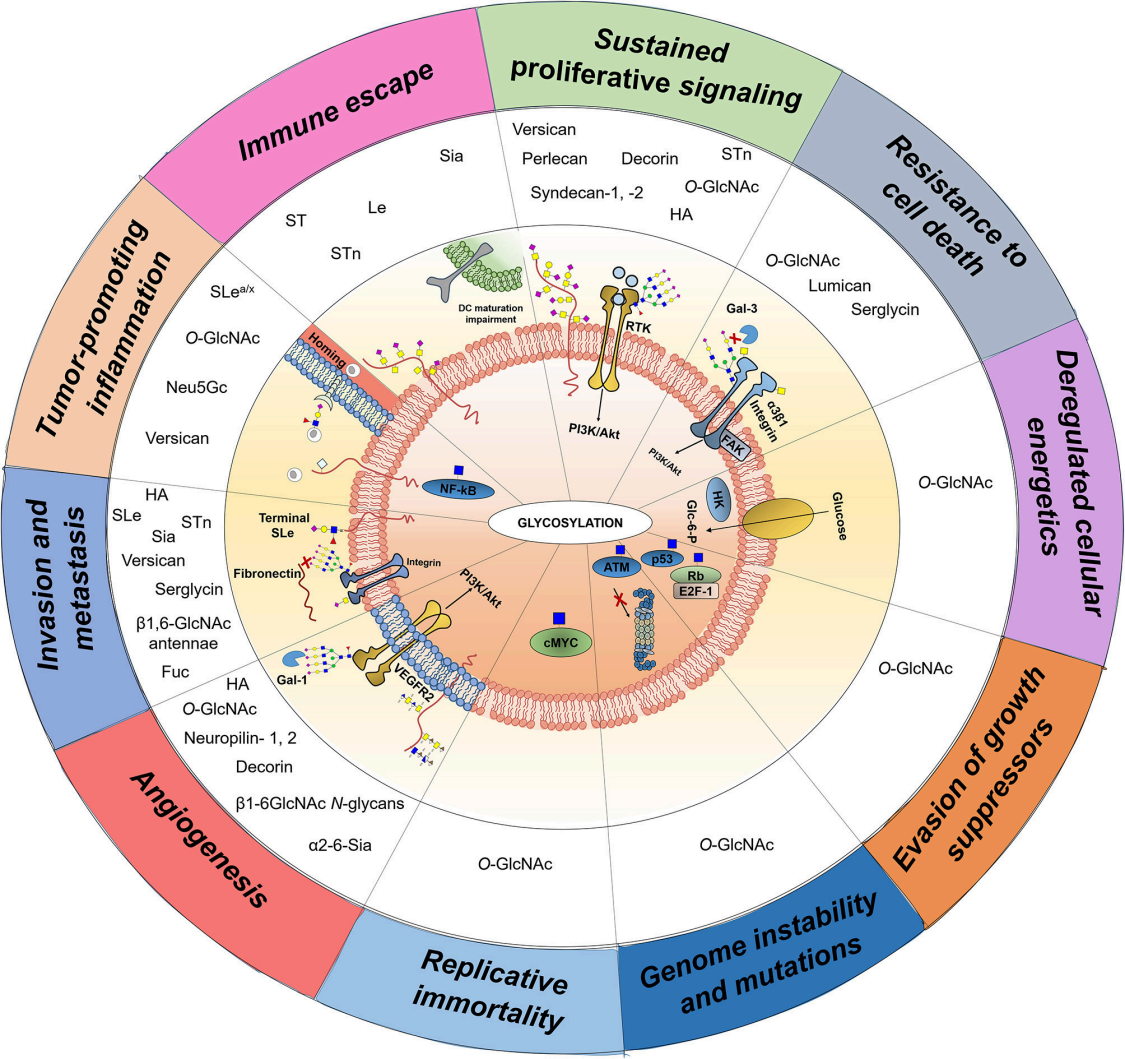
Transversal nature of glycans, glycoproteins, glycan-binding proteins, and proteoglycans throughout the 10 currently accepted cancer hallmarks. Aberrantly expressed glycans (*O*-GlcNAc, Le, SLe, Neu5Gc, Sialic acids (Sia), fucose residues (Fuc), ST, STn), glycoproteins (integrin α3β1, VEGFR2), and proteoglycans (Perlecan, Decorin, Neuropilin-1,-2, Syndecan-1,−2, Hyaluronic acid fragments (HA), Versican, Lumican, Serglycin) are mechanistically involved in cancer hallmarks acquisition. The illustration highlights the most common glycosylation modifications throughout the cancer hallmarks, transmitting the empiric notion that a great number of glycosylation aberrations mostly contribute to disease dissemination through increased angiogenesis and potentiation of invasion and metastasis. Moreover, the post-translational modification β-*O*-N-acetyl-d-glucosamine (O-GlcNAc) emerges as a key regulator of cellular activities through the modulation of signal transduction and protein stabilization. In conclusion, glycans and glycoconjugates are not bystanders to malignant transformation but major players, making then attractive targets to drive molecular-based clinical intervention.

As thoroughly described in the previous sections, tumor stromal cells and ECM components have a preliminary regulatory role in the acquisition of hallmark capabilities, mostly by supplying the soluble factors that drive adaptation or shielding tumor cells from external stress. Glycosylation ads a second level of regulation by governing structural alterations in major receptors, by modifying soluble factors and/or by modulating intracellular kinase cascades (Figure 4). Showcasing this, proliferative signaling is sustained by stromal cells that supply mitogenic factors, while glycosylation promotes growth factor receptor activation and positively regulates intracellular kinases pathways. Besides sustained growth, tumor cells must circumvent programmed cell death to ensure cancer progression. Envisaging this, stromal cells and ECM remodeling provide diffusible paracrine survival factors and non-diffusible survival signals, while offering a physical barrier against pro-apoptotic factors such as chemotherapy. In line with this, glycosylation determines the sensitivity of death receptors to their ligands and drives the initiation of pro-survival cascades, while altering transcription factor activity. Concomitantly, sustained proliferation and programmed cell death evasion culminate in highly energy demanding tumors that establish symbiotic relationships with stromal cells that activate complementary metabolic pathways to buffer and recycle tumor-derived metabolites. Moreover, to sustain growth and survival in the face of hypoxia, HIF-1α strongly regulates glucose metabolism throughout the several biosynthesis pathways, culminating in altered glycosylation precursor expression as well as increased sialylation and *O*-GlcNAcylation toward more aggressive clones. Simultaneously, tumor cells evade growth suppression by abrogating the suppressive role of adhesion complexes with the ECM, mostly by the action of stromal-derived proteolytic enzymes. At the same time, the two canonical suppressors of proliferation p53 and RB are negatively regulated through *O*-GlcNAc modifications. All the above-mentioned events are largely driven by the genomic instability of cancer cells, culminating in advantageous random mutations. This variability thrives much as a consequence of the DNA damage promoted by the mutagenic/oxidative microenvironment indorsed by stromal cells. Also, hypoxia alters the transcription and translation of several DNA damage response and repair genes. In turn, glycosylation modulates DNA damage response pathway components, reinforcing the genomic instability of tumor cells. Interestingly, both the tumor microenvironment and glycosylation have little to do with the replicative immortality of tumor cells, their contribution is mainly based on the indirect regulation of the transcription factor c-MYC and kinase cascades. Importantly, to sustain proliferation and the energetic demands of ever-growing tumors, a pro-angiogenic environment must be established. As such, to ensure neovascularization, stromal cells supply pro-angiogenic factors and upregulate multiple angiogenic pathways culminating in the maturation and survival of endothelial cells. In turn, angiogenic glycans and glycoconjugates alter the affinity of angiogenic factor receptors for their ligands toward a pro-angiogenic phenotype of EC. Advanced stage tumors frequently progress to invasion and metastasis, which is facilitated by the compromised vascular and stromal barriers to tumor cell migration. Moreover, stromal cells can promote EMT in tumor cells and tether these cells to improve colonization at distant sites. Concomitantly, glycosylation changes in tumor cells physically disrupt cell adhesion by upregulating sialylated antigens and *N*-linked β1,6-GlcNAc branches, contributing to increased cellular motility and invasiveness. On the other hand, glycosylation can promote adhesion of tumor cells and still favor the establishment of metastatic colonies. Namely, tumor cells overexpressing SLe^a/x^ are able to roll on the endothelial surface and extravasate into circulation, while arresting its movement in distant locations by interacting with selectins expressed by endothelial cells. Some glycosyltransferases expression also defines the metastatic potential of tumor cells, acting as metastasis suppressors or enablers.

In the meantime, tumor-associated stromal cells contribute to tumor-promoting inflammation by supplying several pro-inflammatory cytokines and chemokines, ultimately driving tumor growth, neovascularization, immune cell recruitment, and glycosyltransferases expression. Furthermore, glycosylation changes not only contribute to the recruitment of circulating lymphocytes into peripheral lymph nodes and inflamed tissues but also regulate the activity of several inflammatory mediators and the polarization of immune cells into immunosuppressor phenotypes. In line with this, the tumor microenvironment increasingly becomes populated with immunosuppressive immune cells. Concomitantly, tumor cells glycosylation, mostly characterized by hypersialylation, promotes immune scape by being simple and “self”-like, by inducing tolerogenic immune cell phenotypes, and by effectively shielding tumor cells from effector immune cells, culminating in tumor progression.

Based on these insights, glycosylation changes reflect not only the genomic, transcriptomic, proteomic, and metabolomic state of cells but also its external microenvironment, making glycosignatures highly context-specific and attractive targets for personalized medicine. Several evidences support the existence of a unique repertoire of glycans associated with disease progression and dissemination, decisively reflecting on virtually all cancer hallmarks (Figure 4). Changes in *O*-GlcNAcylation is the most common glycosylation modification throughout cancer hallmarks, providing a dynamic but highly regulated sensor driving protein stabilization and signal transduction. Sialic acids and, particularly sialylated short-chain *O*-glycans are also amongst the most common structures driving invasion and immune escape, clearly marking more aggressive tumor cell phenotypes. Moreover, the major bulk of glycosylation modifications accompanying malignant transformation seem to contribute to disease dissemination through increased angiogenesis and potentiation of invasion and metastasis. Notwithstanding, little is known about glycosylation contribution to key aspects of neoplastic transformation as the acquisition of genomic instability and replicative immortality, opening an avenue for novel research (Figure 4). The sweet side to this sour end resides on the possibility of exploring the extracellular nature of glycans for targeting tumor and stromal cells using more effective non-invasive tools. As such, we intend to reinforce the need to concentrate efforts to incorporate glycans in broad biomarker panels of different molecular natures, envisaging highly sensitive and specific detection methods for disease monitoring and early intervention. Moreover, by integrating microenvironmental information, glycosignatures will most likely provide the necessary key for designing highly specific cancer ligands envisaging theragnostic applications; thereby allowing guiding therapeutic decision and, more importantly, designing novel therapeutics. Notwithstanding, significant room lays beyond targeted approaches, specially facing the recent advances in glycomics and glycoproteomics. Therefore, it is now possible to engage on a comprehensive study of the glycome and glycoproteome envisaging the necessary glycobiology landscape for intervention. Of note, selectin and galectin antagonists, including glycomimetic compounds, antibodies, aptamers, and peptides are currently in FDA clinical trials and near-clinical trials for the treatment of blood-related cancers and solid tumors metastasis (303). Moreover, the high sensitivity and resolution of new generation mass-spectrometers will allow obtaining structural information almost to a single-cell level, enabling the analysis of exosomes, CTC, and stromal components, which will be crucial for addressing metastatic disease. Overall, we believe that the necessary context has been created to foster more in-depth studies on the glycobiology of tumors and its microenvironment envisaging molecular-based precision medicine and improved patient care.

## Author Contributions

AP and JF wrote the manuscript. AP, MR-S, and RA produced the artwork. MR-S, RA, and LS revised it.

### Conflict of Interest Statement

The authors declare that the research was conducted in the absence of any commercial or financial relationships that could be construed as a potential conflict of interest.
